# Trade Policy and Health: Adding Retrospective Studies to the Research Agenda: Comment on "The Trans-Pacific Partnership: Is It Everything We Feared for Health?"

**DOI:** 10.15171/ijhpm.2016.123

**Published:** 2016-09-10

**Authors:** Chantal Blouin

**Affiliations:** Centre for Interdisciplinary Studies on International Trade and Investment, Institute for Advanced International Studies (HEI), Université Laval, Quebec City, QC, Canada.

**Keywords:** Trade and Investment Policy, Population Health, Social and Political Determinants of Health, Regulatory Chill

## Abstract

Prospective studies of the potential health consequences of trade and investment treaties, such as the Trans-Pacific Partnership, are critical. These studies can make visible to trade policy-makers the potential negative impacts associated to such treaties and can influence the outcomes of such negotiations. However, few researchers have examined retrospectively the consequences of trade agreements. With more than 400 trade agreements and more than 2000 investment treaties currently in force, researchers have a large corpus of agreements to analyse in order to assess not only their potential impacts on health system and population health, but also their actual impacts. This comment suggests some research questions that would benefit from retrospective inquiry.

In the last 20 years, the consequences of trade and investment treaties for population health and for health systems have received an increasing level of attention in the scholarly and policy literature. These publications have examined what could be the consequences of the World Trade Organisation’s (WTO’s) agreements, of regional agreements and plurilateral agreements for population health and/or for health systems. In their article “The Trans-Pacific Partnership: is it everything we feared for health?,” Labonté and colleagues^1^ further contribute to this literature with a rigorous analysis of how this new agreement could affect health in the future.

These prospective analyses are critical to ensure that the potential health consequences of trade policy are taken into considerations by trade policy-makers (see Friel et al,^2^ for a narrative review of this literature). At the national level, where trade policy objectives are first set, health considerations are often absent or ignored by decision-makers. National trade policy’s objectives are mostly defined by the interplay of the economic interests at stake. The influence of economic actors wishing to expand or consolidate export markets or their investments abroad vs. the influence the economic actors advocating to keep protectionist measures in place to ensure domestic production or competitiveness are still the main factors to understand the content of national trade policy.

Analytical work which highlight the health risks associated to trade and investment agreements can support the national health agencies involved in inter-sectoral work to influence their trade colleagues. It can also inform the work of health professionals, their associations and various organisations advocating for health-promoting public policies at the national and global level. Such prospective analysis can clarify unintended consequences of the provisions of the agreements, it can shed light on the costs and risks that were not integrated into the trade negotiators calculation of the “trade-offs” at stake.

Trade negotiations are still viewed by negotiators as mutual exchanges of concessions (“you open your agricultural markets for my producers, in exchange I will open my telecommunications markets to your providers”). If the costs in terms of higher prices for medicines, or reduced capacity to regulate to protect and promote health are not made visible, they will not even be part of the “grand bargain” of trade negotiations. Making these costs visible is a necessary, if not sufficient, step toward tackling the political determinants of health (see Ottersen et al^3^).

However, one type of research is not sufficiently conducted. Few have examined retrospectively the impact of trade agreements. With more than 400 trade agreements and more than 2000 investment treaties currently in force, researchers now have a large corpus of agreements to analyse in order to assess not only their potential impacts on health system and population health, but also their actual impacts.

To my knowledge, this type of retrospective inquiry has been limited to three areas:

1. The impact of greater economic integration, which is partially linked to trade agreements, on income and poverty as key determinants of health (see a review of that literature in Blouin et al^4^);

2. The impact of intellectual property provisions in trade agreements on the price of medicines,^5,6^ and

3. The legal consequences of trade disputes that have involved human health issues (see for example McGrady^7^).

Several other empirical questions could be explored with retrospective studies. Let’s take the example of the risk of regulatory chill. It refers to situations where “governments become more reluctant to enact new policy for fear of being sued.”^1^ This is a real concern from a population health perspective, as it could mean that the most effective or promising measures are avoided because officials do not want to take a risk that this measure be challenged in a trade or investment disputes. This concern is especially salient in the case of investor-statement dispute settlement, where private organisations can directly challenge policies that they deemed to violate trade and investments treaties.

To what extent policy-makers in national agencies have decided against, or modified certain policy and regulations because they feared that it would lead to trade disputes? To what extent local and national authorities integrate an analysis of the exposure risks? Even when policy-makers are made aware of a potential risk, ie, that a measure could be challenged by foreign investors, how much this information influence their decision-making? Can we identify political or economic conditions that are more likely to lead to regulatory chill in health policy-making?

Another question relates to the broader policy-making context. Are trade agreements being used by private industry or other opponents of public health measures as a political resource to block or modify legislation or regulation? Many health-promoting public policies under consideration by national and local governments have been opposed by private industry using traditional domestic lobbying and advocacy strategy. The numerous failed attempts by state legislatures to impose taxes on sweetened beverages in the United States illustrate the influence of such opposition.^8^ To what extent trade and investment treaties are seen and used as a resources by industry to oppose a specific policy? Do traditional advocacy and lobbying tools remain the main channels for influence?

Answering these types of question will require for political scientists and other social scientists to undertake in-depth case studies and to use qualitative research methods such as process tracing which allows to examine the policy process with a view to understand the causal sequences leading to particular policy decisions.^9^

On the other hand, other retrospective inquiries will require quantitative methods, in order to ascertain whether trade agreements have had positive or negative impacts on the various determinants of health. For example, has the liberalisation of agricultural trade improve or worsen food security? Facilated or created obstacles to the adoption of healthier diets? Similar questions can be asked about the impact of trade agreements on national health systems. For instance, has the liberalisation of trade in services led to increased privatisation of health services and insurance? Or led to other transformations in the provision and financing of healthcare services? In sum, a diversity of research methods and disciplinary expertise will be require to investigate the multiple linkages between trade and health.

A retrospective research program of the impact of trade and investment treaties on national and sub-national policy-making would complement the existing work on the potential risks and benefits of such treaties. Such research program can help identify the priority areas to focus on to ensure that trade and investment treaties contribute to improve population health, and rather than increasing the price of essential medicines, reducing access to services, or reducing policy space for health-promoting public policies.

## Ethical issues

Not applicable.

## Competing interests

Author declares that she has no competing interests.

## Author’s contribution

CB is the single author of the paper.
